# Constitutive Model of Secondary Annealing Behavior of Cu-Cu Joints in Cu/SiO_2_ Hybrid Bonding

**DOI:** 10.3390/ma18225152

**Published:** 2025-11-13

**Authors:** Yiming Hao, Si Chen, Chao Li, Zejian Chen, Fei Qin, Pei Chen, Renjie Tian, Ziyang Li

**Affiliations:** 1 Institute of Electronics Packing Technology and Reliability, Beijing University of Technology, Beijing 100124, China; yiminghao@emails.bjut.edu.cn (Y.H.);; 2 Science and Technology on Reliability Physics and Application of Electronic Component Laboratory, China Electronic Product Reliability and Environmental Testing Research Institute, Guangzhou 511300, China; 3 National Center for Advanced Packaging Co., Ltd., Wuxi 214112, China

**Keywords:** secondary annealing, Cu/SiO_2_ hybrid bonding, constitutive model, nanoindentation, microstructure

## Abstract

In this study, the stress–strain constitutive models of Cu-Cu joints in hybrid bonding after primary and secondary annealing were determined using nanoindentation experiments and finite element inverse analysis, and the correlation mechanism between the microstructure and macroscopic mechanical properties in hybrid bonding Cu-Cu joints during secondary annealing was revealed. The 350–400 °C secondary annealing facilitates recrystallization–grain growth, increasing grain size from 0.62 μm after primary annealing to 0.71 μm, accompanied by a 12% reduction in kernel average misorientation (KAM) values. This process enhances interface non-planarization and optimizes bonding quality. Concurrently, the secondary annealed Cu-Cu joints exhibit a softening effect, manifested by decreasing trends in elastic modulus (131.02 → 118.98 GPa), hardness (1.78 → 1.51 GPa), and yield strength (70.52 → 56.12 MPa), primarily attributed to the Hall–Petch effect and residual stress release. Notably, the yield strength of secondary annealed Cu-Cu joints demonstrates 31.0% and 68.5% enhancements compared to TSV-Cu (42.83 MPa) and bulk Cu (33.3 MPa), respectively.

## 1. Introduction

Cu/SiO_2_ hybrid bonding has emerged as a critical technology for ultra-high-density packaging; it has been demonstrated to scale below a 1 μm pitch and exhibits superior electrical and mechanical properties. Owing to its promising applications in advanced electronic products in the future, Cu/SiO_2_ hybrid bonding has drawn a lot of attention in recent years [1,2,3].

An ideal Cu-Cu joint relies on the thermal expansion of Cu and on atom diffusion at the bonding interface during the annealing process. When the annealing temperature is lower than 300 °C, there is no obvious grain growth in the Cu-Cu joint, leaving sharp interfaces in the original bonding surfaces [4,5,6]. Moreover, due to differences in the CTE of various materials between Si, Cu, and dielectric materials, deformation and residual stresses are introduced at the interfaces during annealing, leading to the formation of microcracks and microvoids at the Cu/Cu and Cu/SiO_2_ interfaces, which significantly increase the risk of potential delamination. Juang [7] and Shie [8] found that different annealing temperatures and times affect the size of voids and the bonding rate at the Cu-Cu joint interface. Panchenko [9,10] studied the changes in Cu grains after different reliability tests and found that Cu grains become significantly coarser after storage at high temperatures of 300–400 °C.

The mechanical properties of Cu-Cu joints exhibit corresponding alterations alongside the evolution of their microstructures (e.g., coarser grain, interfacial void redistribution). Lim [11] observed that the shear strength can increase 77% in the Cu joints when the original bonding interface is eliminated. By utilizing surface activation technology to engineer Cu bonding surfaces, Kang [12] realized a Cu-Cu interface at 200 °C featuring robust atomic diffusion, pronounced grain growth, and reduced microvoid formation, with interfacial bonding strength demonstrating a sixfold enhancement. Juang [4] observed that increasing the temperature led to an improvement in the shear strength of the Cu joints. Ni [13] discovered that introducing Cu nanoparticles into Cu-Cu joints modified the microstructural evolution pattern at the interface, resulting in a 64.9% enhancement of peel strength.

Nevertheless, traditional macro–micro tensile [14] and four-point bending [15] experimental protocols require sample preparation into rodlike or flaky structures, rendering them unsuitable for micro–nano scale Cu joint structures in hybrid bonding. This approach lacks in situ characterization methods for mechanical testing at the micro–nano scale to obtain intrinsic material parameters such as elastic modulus, hardness, and yield strength. The in situ measurement data of yield stress for Cu-Cu joints have not been systematically reported to date, particularly after the annealing process with grain size dependency analysis, thereby preventing quantitative evaluation of the mechanical characteristics in Cu-Cu joint interfaces. Actually, the failure mechanism of Cu-Cu joints is closely related to mechanical parameters such as elastic modulus and yield strength. The elastic modulus affects the ability to resist deformation, while the yield strength dominates its plastic response. Therefore, it is necessary to establish the relationship between the Cu-Cu joints’ microstructure and mechanical properties. Inspired by Pshyk’s exploration of in situ nanomechanical measurement techniques [16], combining nanoindentation to obtain parameters like elastic modulus, yield stress, and hardness for Cu-Cu joints emerges as a primary approach.

In this study, a secondary annealing treatment was applied to Cu-Cu bonded interfaces to promote the coarsening of interfacial grains and enhance microstructural homogeneity. Nanoindentation experiments integrated with finite element inverse analysis were conducted to establish the elastoplastic constitutive relationships of Cu-Cu joints for both primary and secondary annealing conditions, while their microstructural evolution was quantitatively characterized. The variations in interface hardness, elastic modulus, and yield strength were systematically investigated through comparative analysis of primary and secondary annealing processes, aiming to provide theoretical guidance for improving the thermomechanical reliability of Cu/SiO_2_ hybrid bonding structures.

## 2. Experiments

### 2.1. The Preparation of Cu/SiO_2_ Hybrid Bonding Structures

**Figure 1:**
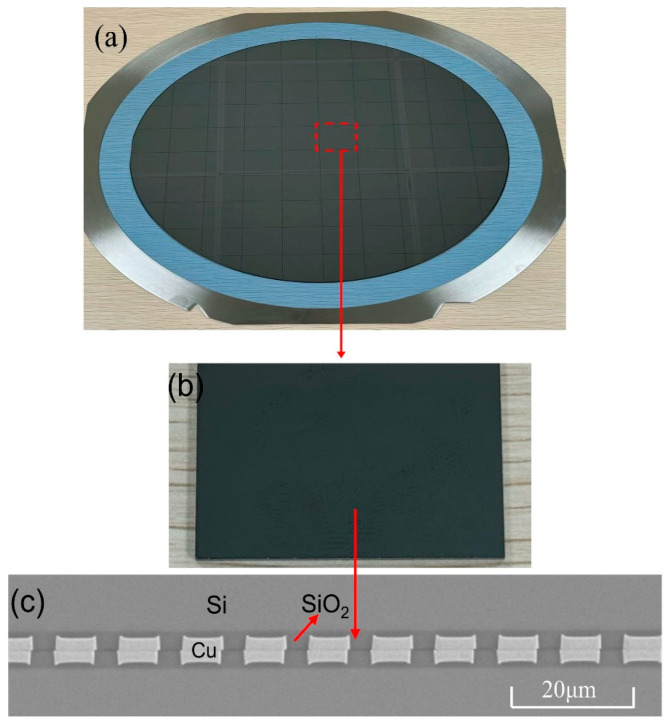
(**a**) The 12-inch bonding wafer, (**b**) 3 × 3 mm chips, (**c**) SEM image of the cross-section of the Cu/SiO_2_ hybrid bonding structure.

The fabrication process of 12-inch wafers for Cu-Cu joints samples is as follows: a 3.5 µm silicon oxide (SiO_2_) layer was deposited on the wafer surface via plasma-enhanced chemical vapor deposition (PECVD), followed by chemical mechanical polishing (CMP) to reduce the SiO_2_ layer thickness to 3 µm. Pad patterning was achieved through photolithography, and plasma etching was employed to create pad recesses in the SiO_2_ layer. Electroplating was then utilized to fill the recesses with Cu pads. Subsequently, two wafers were bonded at room temperature, followed by annealing at 300 °C to finalize the hybrid bonding, as shown in Figure 1a. Then, the wafer was diced into 3 × 3 mm chips, as shown in Figure 1b. The cross-sectional view of the sample is illustrated in Figure 1c; the Cu pad has a diameter of 6 μm and a pitch of 10 μm.

### 2.2. Secondary Annealing

The secondary annealing was performed in a GSL-1750 single-zone tube furnace using high-purity argon gas (99.9999% purity) as the protective atmosphere, with a flowing argon environment maintained at atmospheric pressure (total flow rate: 100 mL/min). The samples were heated from room temperature (25 °C) to the target temperature at a heating rate of 5 °C/min, held at the annealing temperature for 30 min, and then naturally cooled to room temperature. Considering that Cu-Cu joints require a high temperature of 400 °C for sufficient atomic diffusion [17,18,19], 350 °C and 400 °C were selected as the secondary annealing temperatures.

### 2.3. Mechanical Properties Testing

**Figure 2:**
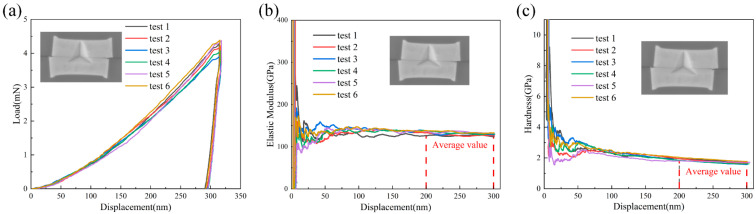
(**a**) Load–displacement curve, (**b**) modulus–displacement curve, (**c**) hardness–displacement curve.

Nanoindentation tests were conducted on the cross-section of Cu-Cu in the Cu/SiO_2_ hybrid bonding structure using a G200 Nano Indenter equipped with a Berkovich diamond indenter (Agilent Corporation, Santa Clara, CA, USA). The continuous stiffness method (CSM) [20] was employed to obtain load/displacement curves, as well as the variations in elastic modulus and hardness with indentation depth. During the test, the maximum indentation depth was set to 300 nm, and the strain rate was maintained at 0.05 s^−1^. Six Cu-Cu joint test points were selected for indentation in each experimental group. Figure 2 presents the results after the first annealing of the sample. Both elastic modulus and hardness values stabilize within the 200–300 nm depth range, and a similar trend was also observed for the secondary annealed samples. Consequently, the stable values of elastic modulus and hardness within this depth range were adopted as the final experimental results.

### 2.4. Microstructural Characterization

An EBSD detector (Symmetry, Oxford Instruments, High Wycombe, UK) was used to analyze the microstructure of the annealed Cu-Cu joints, with the voltage set to 20 kV and the acceleration current set to 5 nA. During the sample preparation procedure, silicon carbide abrasive paper, 1 μm diamond polishing slurry, and 0.05 μm alumina polishing fluid were utilized to obtain a flat Cu-Cu joint cross-section surface. The high-quality surface of Cu-Cu joints is a prerequisite to obtain EBSD patterns successfully since any contamination or residual deformation will severely destroy the EBSD patterns [21]. Therefore, ion milling (Leica EM TIC 3X, Leica Microsystems, Wetzlar, Germany) was performed to remove the residual stress layer of the cross-section of Cu-Cu joints. The Ar ion source was applied by setting the milling voltage of 6 kV for 20 min in the first step and setting 5 kV for 20 min in the second step. Finally, Channel 5 software was used to analyze the microstructure of the Cu-Cu joints based on the EBSD images obtained.

## 3. Inversion Algorithm and Constitutive Modeling

**Figure 3:**
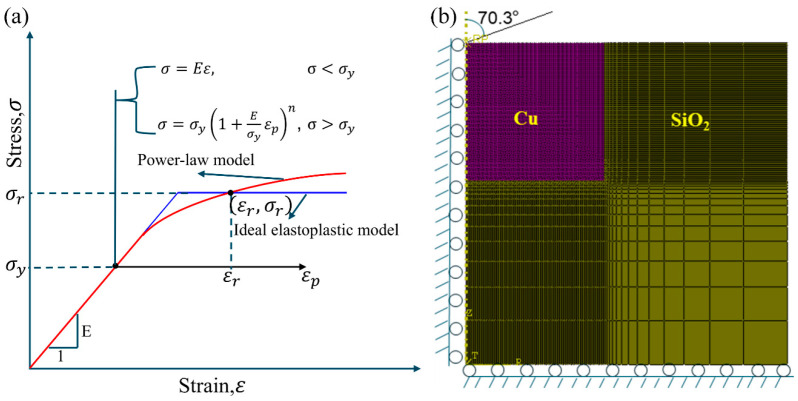
(**a**) Elastic–plastic constitutive model of metal materials, (**b**) finite element model.

Similarly to most of the metals, a power law constitutive model was used to invent the stress–strain relationship of Cu-Cu joints, as shown in Figure 3a and Equation (1):

(1) $$\sigma =\left\{\begin{array}{cc} E\varepsilon & \sigma \leq \sigma_{y}\\ \sigma_{y}{\left(1+\frac{E}{\sigma_{y}}\varepsilon_{p}\right)}^{n} & \sigma >\sigma_{y} \end{array}\right.$$

where *E* is the elastic modulus, *σ_y_* is the yield strength, $\varepsilon_{p}$ is the plastic strain, and *n* is the hardening exponent. The stress–strain relationship is shown by the red curve in Figure 3a, which intersects with the blue curve representing the ideal elastic–plastic constitutive model at a special point ($\varepsilon_{r},\sigma_{r}$), where $\varepsilon_{r}$ is the characteristic strain and $\sigma_{r}$ is the characteristic stress. For materials with the same elastic modulus, consistent load–displacement curves in nanoindentation tests can be achieved if their stress–strain curves pass through this critical point [22].

**Figure 4:**
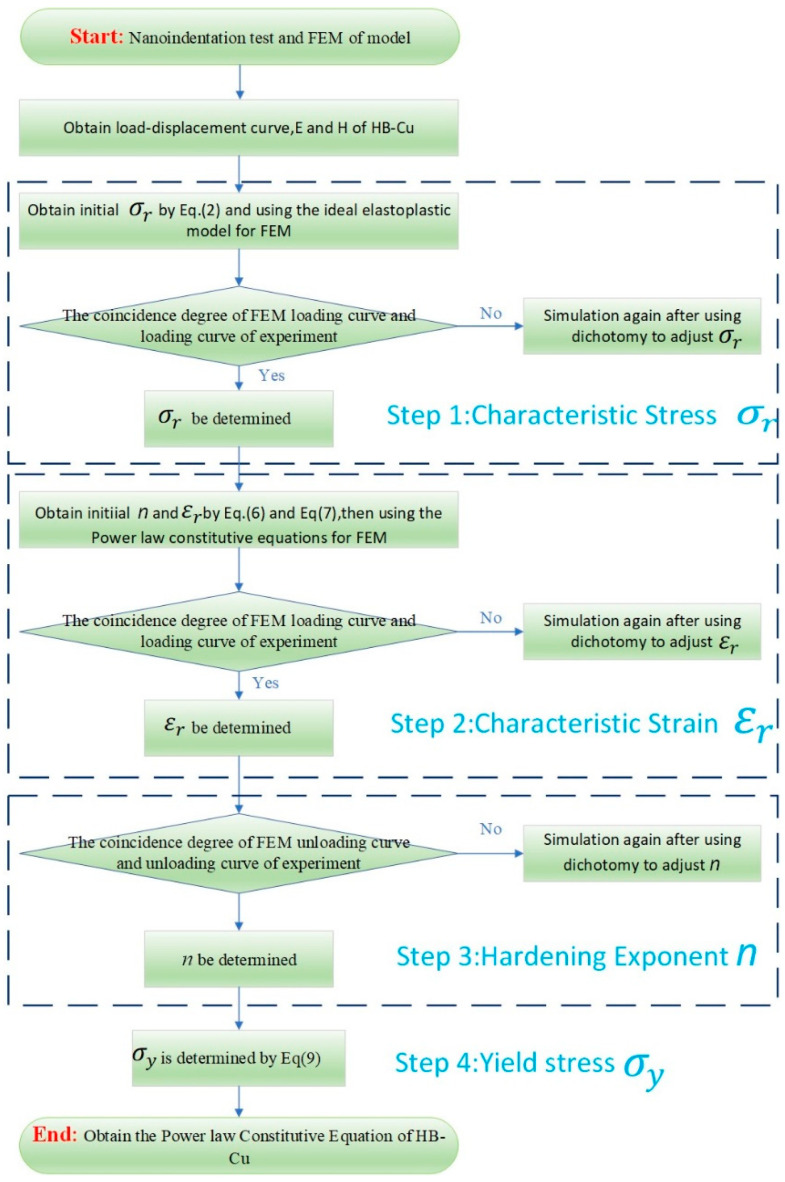
Flowchart for determining the power law type constitutive relationship of Cu-Cu joints.

The commercial software ABAQUS 2021 was employed to calibrate the constitutive model of Cu-Cu joints through finite element inverse analysis. A two-dimensional axisymmetric finite element model was established, as shown in Figure 3b. The interfaces between Cu and SiO_2_ were connected via shared nodes. To ensure inversion accuracy, the displacement and boundary conditions of the model were consistent with experimental settings: symmetric constraints were applied to the left and bottom edges of the model, while vertical constraints were imposed at the bottom. The element type CAX4R was adopted, totaling 10,464 elements. Following the equivalent projection principle [23], the Berkovich indenter was modeled as a conical indenter with a half-cone angle of 70.3°. The indenter was treated as a rigid material, with a contact friction coefficient of 0.1 defined between the indenter and the Cu/Cu surface. The elastic modulus and Poisson’s ratio of SiO_2_ were set to 73 GPa and 0.17, respectively [24], while the elastic modulus of the Cu-Cu joint was obtained from nanoindentation experiments, as given in Table 1, and its Poisson’s ratio was set to 0.3. Figure 4 illustrates the algorithm flowchart to determine the power law constitutive relationship of the Cu-Cu joints.

**Step 1:**

The initial characteristic stress σ_r_ is calculated using Equation (2) proposed by Antunes et al. [25]:

(2) $$\frac{E_{r}}{H}=0.231\left(\frac{E_{r}}{\sigma_{r}}\right)+4.910$$

where *H* is the hardness of the Cu obtained through nanoindentation testing and $E_{r}$ is the shear modulus and can be obtained using Equation (3).

(3) $$\frac{1}{E_{r}}=\frac{1-\nu^{2}}{E}+\frac{1-{\nu_{i}}^{2}}{E_{i}}$$

where E is the elastic modulus of Cu obtained experimentally and v is the Poisson’s ratio, taken as 0.3; the elastic modulus *E*_i_ and Poisson’s ratio *v*_i_ of the diamond indenter are 1141 GPa and 0.07, respectively [26].

The hardening exponent *n* is initially set to 0 to eliminate its influence on inversion results. Through iterative computation using Equation (4) [27], the characteristic stress *σ*_*r*_ is updated until the relative error between the maximum simulated load $P_{\max}^{FEM}$ obtained from finite element inversion and the experimentally measured maximum load $P_{\max}^{EXP}$ falls below 0.5%, at which stage the final value of *σ*_*r*_ is determined.

(4) $$\sigma_{r}\left(i+1\right)=\sigma_{r}\left(i\right)\frac{P_{\max}^{\mathrm{EXP}}}{P_{\max}^{\mathrm{FEM}}}$$

where $\sigma_{r}(i)$ and $\sigma_{r}(i+1)$ represent the characteristic stresses at the i-th and i + 1-th iterations, respectively.

**Step 2:**

**Figure 5:**
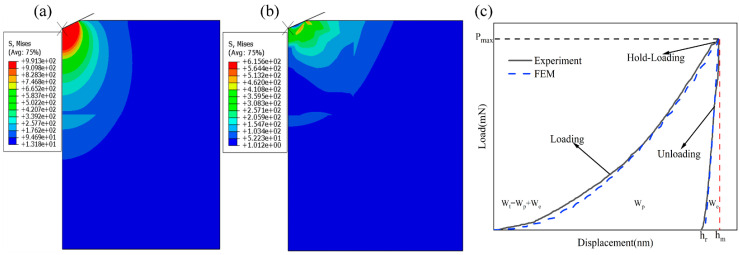
(**a**) The von Mises stress contour map at maximum load depth, (**b**) von Mises stress contour map after unloading, (**c**) load–displacement curves of nanoindentation experiments and finite element simulation curve.

By substituting the characteristic stress *σ_r_* into the dimensionless function expressions (5) and (6) proposed by Dao et al. [28], the hardening exponent *n* can be explicitly determined.

(5) $$\frac{W_{P}}{W_{T}}=1.61217\left\{1.13111-1.74756^{\left[-1.49219{\left(\frac{h_{r}}{h_{m}}\right)}^{2.535334}\right]}-0.075187{\left(\frac{h_{r}}{h_{m}}\right)}^{1.135826}\right\}$$

where $W_{p}$ is the area enclosed between the loading and unloading curves in Figure 5c, $W_{e}$ is the area under the unloading curve, and $W_{T}$ is the total work done by the indenter. $h_{r}$ is the maximum indentation depth during loading and $h_{m}$ is the residual depth after complete unloading.

(6) $$\frac{h_{r}}{h_{m}}=A{\left[\ln(\frac{\sigma_{r}}{E_{r}})\right]}^{3}+B{\left[\ln(\frac{\sigma_{r}}{E_{r}})\right]}^{2}+C[\ln(\frac{\sigma_{r}}{E_{r}})]+D$$

A = 0.010100*n*^2^ + 0.0017639*n* − 0.0040837
B = 0.14386*n*^2^ + 0.018153*n* − 0.088198
C = 0.59505*n*^2^ + 0.034074*n* − 0.65417
D = 0.58180*n*^2^ − 0.088460*n* − 0.67290

Owing to the parametric independence of *σ_r_* and *ɛ_r_*, their values remain unaffected by variations in *n* [29]. The initial estimate of the characteristic strain *ɛ_r_* can be determined by solving the governing Equation (7) established by Lee et al. [30]:

(7) $$\varepsilon_{r}=\exp\left(\frac{166.7}{E_{r}/\sigma_{r}+177.3}-3.91\right)$$

The elastoplastic constitutive equation, defined by the initial characteristic strain $\varepsilon_{r}$, the derived characteristic stress $\sigma_{r}$, and the hardening exponent *n*, was implemented in ABAQUS for finite element analysis. Through iterative computation using Equation (8), the characteristic stress ɛ_*r*_ is updated until the relative error between the maximum simulated load $P_{\max}^{FEM}$ obtained from finite element inversion and the experimentally measured maximum load $P_{\max}^{EXP}$ falls below 0.5%, at which stage the final value of ε_*r*_ is determined.

(8) $$\varepsilon_{r}(i+1)=\varepsilon_{r}(i)\frac{F_{\max}^{\mathrm{FEM}}}{F_{\max}^{\mathrm{EXP}}}$$

The von Mises stress distributions of the Cu-Cu joints at the maximum loading depth and after unloading are illustrated in Figure 5a and Figure 5b, respectively. The maximum values appear directly below the indenter.

**Step 3:**

According to the model proposed by Antunes [24], the unloading segment of the load/displacement curve is governed by the parameter *n*. To optimize *n*, its value was iteratively adjusted to maximize the alignment between the simulated and experimental curves. This involved calculating the slopes at the local maximum and minimum points of the unloading segment to evaluate curve consistency. When the slope error between the two curves reached 0.07% (as shown in Figure 5c), the parameter *n* was finalized.

**Step 4:**

Substituting the final values of *σ_r_*, *ε_r_*, and *n* into Equation (9) allows determination of the yield strength for Cu-Cu joints, from which the complete power law constitutive equation of the bonded system is derived.

(9) $$\sigma_{r}=\sigma_{y}{\left(1+\frac{E}{\sigma_{y}}\varepsilon_{r}\right)}^{n}$$

## 4. Results and Discussion

### 4.1. Constitutive Behavior of Cu-Cu Joints

**Figure 6:**
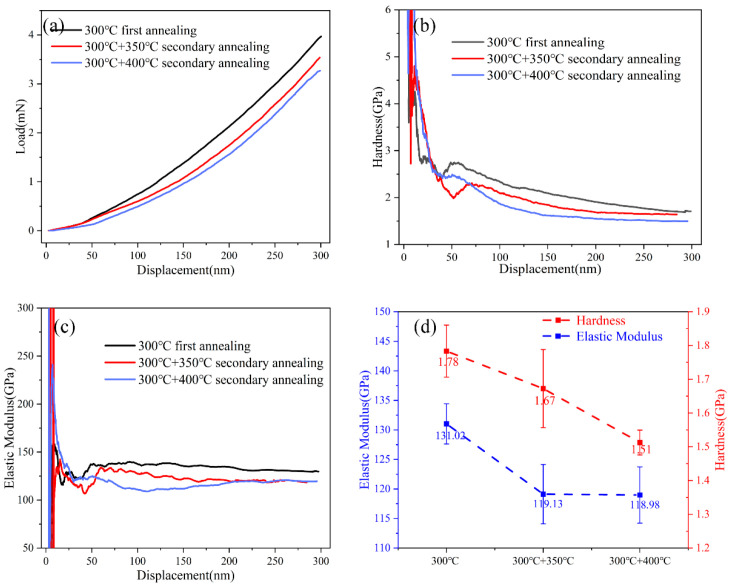
Nanoindentation curves under different conditions: (**a**) load–displacement curve, (**b**) hardness–displacement curve, (**c**) modulus–displacement curve, (**d**) hardness and modulus distribution.

Figure 6a displays the nanoindentation displacement–load curves of Cu-Cu bonds after primary annealing at 300 °C and subsequent secondary annealing at 350 °C and 400 °C. Under the same indentation depth, the specimens subjected to secondary annealing require significantly lower loads compared to those after primary annealing. Additionally, Figure 6b,c illustrate the variations in hardness and elastic modulus with displacement, respectively. The mechanical parameter responses stabilize when the indentation depth reaches 200–300 nm. The stabilized hardness and elastic modulus values are shown in Figure 6d. Compared to the primarily annealed specimens, secondary annealing at 350 °C and 400 °C reduces the elastic modulus from 131.2 GPa to 119.13 GPa and 118.98 GPa, and lowers the hardness from 1.78 GPa to 1.67 GPa and 1.51 GPa, respectively. This indicates that Cu-Cu bonds exhibit softening behavior after secondary annealing, and this softening effect becomes more pronounced as the secondary annealing temperature increases from 350 °C to 400 °C.

**Figure 7:**
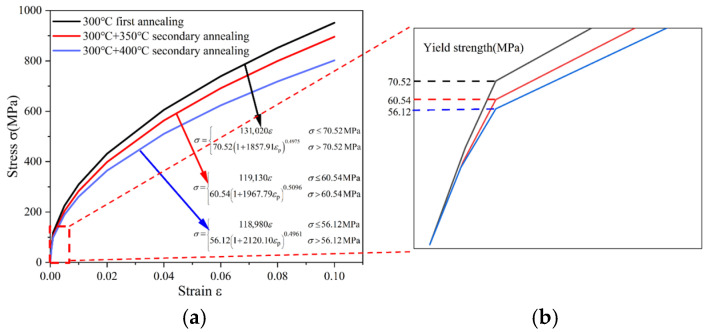
(**a**) Power law constitutive behavior of Cu-Cu joints in different states, (**b**) local enlarged view.

The power law constitutive relationships under different states are shown in Table 1 and Figure 7. It can be seen that, compared with the primary annealed specimens, the yield strength of Cu-Cu joints decreased from 70.52 MPa to 60.54 MPa and 56.12 MPa after secondary annealing at 350 °C and 400 °C, respectively. This indicates that secondary annealing leads to a reduction in the yield strength of Cu-Cu joints, and the decrease becomes more significant as the secondary annealing temperature increases. The decline in yield stress alleviates the risk of stress concentration at the bonding interface.

### 4.2. Microstructural Evolution of Cu-Cu Joints

**Figure 8:**
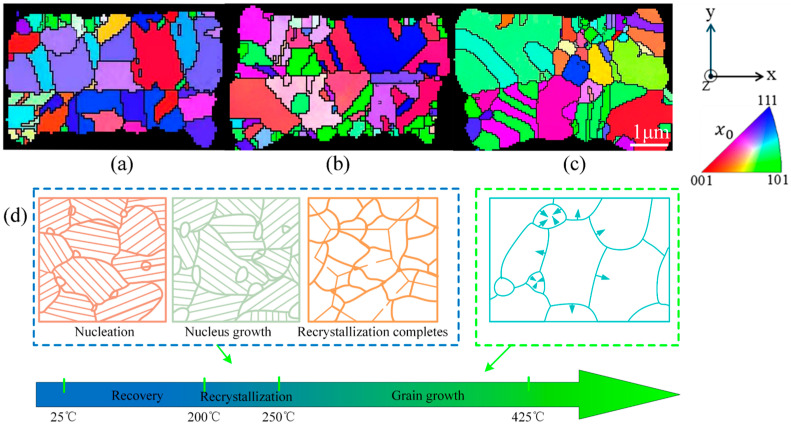
EBSD images of Cu-Cu joints in different states: (**a**) 300 °C first annealing, (**b**) 300 °C + 350 °C secondary annealing, (**c**) 300 °C + 400 °C secondary annealing, (**d**) microstructural evolution of Cu.

Figure 8a–c presents the EBSD maps of the Cu-Cu joints after first annealing at 300 °C and secondary annealing at 350 °C and 400 °C. For the first annealed sample shown in Figure 8a, the average Cu grain size was 0.62 μm. After secondary annealing at 350 °C, the average grain size increased to 0.67 μm (Figure 8b), and further reached 0.71 μm following secondary annealing at 400 °C (Figure 8c). These results demonstrate that secondary annealing triggers grain growth, and the grain growth effect becomes more pronounced as the secondary annealing temperature rises (from 350 °C to 400 °C). This grain growth is associated with the microstructural evolution of Cu-Cu joints during annealing [31]. When the annealing temperature exceeds the recrystallization temperature of copper (200–250 °C [32]), the Cu microstructure undergoes three evolutionary stages, as illustrated in Figure 8d.

Recovery stage: As the temperature increases, strain accumulates within the Cu in the form of deformation energy. This stored energy becomes the driving force for subsequent recrystallization.

Recrystallization stage: When the temperature rises further into the recrystallization temperature range of Cu (200–250 °C), nucleation initiates at the triple points of grain boundaries and gradually grows.

Grain growth stage: Elevated temperatures enhance grain boundary mobility, leading to macroscopic grain coalescence and growth.

**Figure 9:**
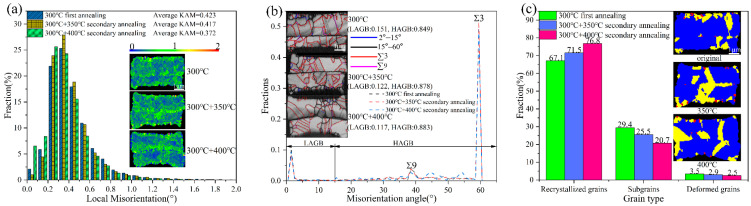
(**a**) Distribution of local orientation differences in Cu-Cu joints in different states, (**b**) distribution and proportion of grain boundary types in Cu-Cu joints in different states, (**c**) recrystallisation distribution of Cu-Cu joints in different states.

This grain growth process is accompanied by a reduction in grain misorientation and a decrease in dislocation density. Figure 9a presents the kernel average misorientation (KAM) evolution of Cu-Cu joints after the first annealing at 300 °C and secondary annealing treatments at 350 °C and 400 °C. Compared to the first annealed sample, the KAM values of Cu-Cu joints decreased from 0.423 to 0.417 and 0.372, respectively, following secondary annealing at 350 °C and 400 °C. This indicates that the deformation and dislocations within the grains of the Cu-Cu joints were partially released after secondary annealing, with a more pronounced reduction observed at 400 °C than at 350 °C.

**Figure 10:**
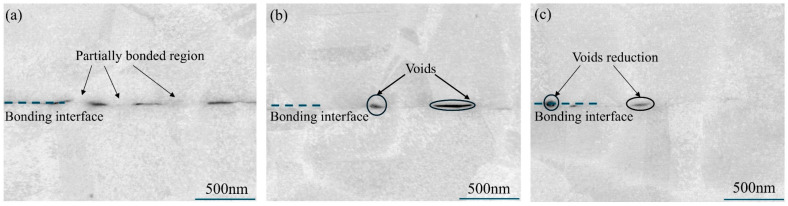
Cross-sectional SEM image of Cu-Cu joints bonding interface: (**a**) 300 °C, (**b**) 300 °C + 350 °C, (**c**) 300 °C + 400 °C.

The grain boundary distribution of Cu-Cu joints is shown in Figure 9b. Compared to the first annealed sample, the proportion of high-angle grain boundaries (HAGBs) in Cu-Cu joints increased from 0.849 to 0.878 and 0.883 after secondary annealing at 350 °C and 400 °C, while the low-angle grain boundaries (LAGBs) proportion decreased from 0.151 to 0.122 and 0.117, respectively. Additionally, the grain boundary distribution reveals that the interface of Cu-Cu joints after the first annealing appeared relatively flat, suggesting limited grain intergrowth at the interface. However, secondary annealing significantly improved the non-planarity of the Cu-Cu joint interface, indicating enhanced grain intergrowth at the interface, with more pronounced effects at 400 °C than at 350 °C. Grain intergrowth was observed to predominantly occur at triple junctions, which was also reported by Panchenko et al. [10] after a 6 h hold at 400 °C in Cu-Cu joints. In addition, Figure 10 shows the void distribution in the Cu/Cu bonding after the first annealing and secondary annealing at 350 °C and 400 °C. After the first annealing at 300 °C, numerous voids were observed at the Cu/Cu joint bonding interface, with only localized regions achieving effective bonding. Following secondary annealing at 350 °C, the interfacial contact area gradually expanded, and the void area decreased. After secondary annealing at 400 °C, only a small number of voids remained at the Cu/Cu joint interface, indicating a significant improvement in the bonding interface quality of the Cu/Cu joints after secondary annealing. Consistent phenomena were observed in the study by Juang et al. [4].

Figure 9c illustrates the recrystallization distribution of Cu-Cu joints. Compared to the first annealed sample, the proportion of recrystallized grains in Cu-Cu joints increased from 67.1% to 71.5% and 76.8% after secondary annealing at 350 °C and 400 °C, respectively, while the proportion of subgrains decreased from 29.4% to 25.5% and 20.7%. This demonstrates that during secondary annealing, subgrains evolved into recrystallized grains surrounded by high-angle grain boundaries (HAGBs) through the absorption of dislocations and grain boundary migration.

### 4.3. Micro–Macro Responses of Cu-Cu Joints

**Figure 11:**
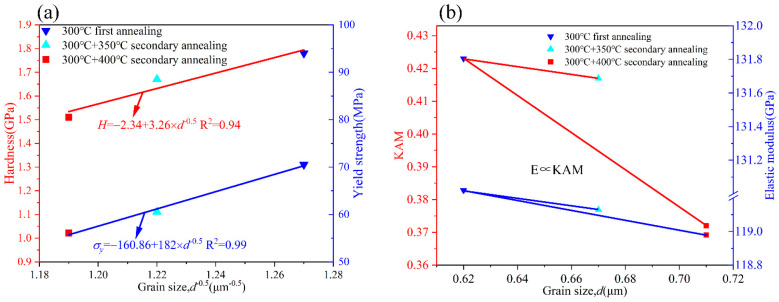
(**a**) Distribution of hardness and yield strength with the square root of varying grain sizes, (**b**) distribution of KAM and elastic modulus with varying grain sizes.

Annealing treatment induces microstructural evolution in Cu-Cu joints and significantly alters their macroscopic mechanical properties. Figure 11a illustrates the distribution of hardness and yield strength across samples with varying grain sizes. It is evident that both yield strength and hardness exhibit a decreasing trend as the grain size increases. Consistent with the Hall–Petch relationship (Equations (10) and (11)), yield strength and hardness are inversely proportional to the square root of the average grain size *D*, demonstrating that these mechanical parameters are primarily governed by grain size. In addition, the thermal activation energy provided during secondary annealing can drive vacancy diffusion towards dislocation lines. When vacancies diffuse to and are absorbed by dislocation cores, this effectively promotes dislocation climb and rearrangement, accelerating dislocation annihilation and thereby inducing softening behavior in the material.

(10) $$H=H_{0}+\frac{K_{1}}{\sqrt{D}}$$

(11) $$\sigma_{y}=\sigma_{y0}+\frac{K_{2}}{\sqrt{D}}$$

In these models, *H*_0_ and σ_y0_ are governed by the initial dislocation density, while *K*_1_ and *K*_2_ represent material-specific coefficients. The Cu-Cu joints subjected to secondary annealing achieve a yield strength of 56.12 MPa, demonstrating superior resistance to plastic deformation compared to TSV-Cu [29] (42.83 MPa) and bulk Cu [33] (33.3 MPa).

Figure 11b illustrates the distribution of elastic modulus and KAM (Kernel Average Misorientation) values across samples with varying grain sizes. The elastic modulus is primarily controlled by residual stress, with its mechanism rooted in the release of the residual stress induced by grain growth during annealing. Budiman et al. [34] observed a reduction in internal residual stress after annealing, which is further supported by the decrease in KAM (Kernel Average Misorientation) values shown in Figure 11b, confirming residual stress relaxation in Cu-Cu joints after secondary annealing. This stress reduction is identified as one of the key factors contributing to the decline in elastic modulus. Similar patterns were also reported in the work by Li et al. [29], reinforcing the critical role of residual stress in modulating elastic properties.

## 5. Conclusions

In this study, the stress–strain constitutive models of Cu-Cu joints in hybrid bonding after primary and secondary annealing were determined using nanoindentation experiments and finite element inverse analysis, and the influence mechanism of microstructural evolution on its macroscopic mechanical behavior during annealing was revealed. The conclusions are as follows:

(1) The secondary annealing processes with temperature of 350 °C and 400 °C enhance the softening effect of Cu-Cu joints, as shown by synchronized reductions in elastic modulus (131.02 GPa → 119.13 GPa/350 °C → 118.98 GPa/400 °C), hardness (1.78 GPa → 1.67 GPa/350 °C → 1.51 GPa/400 °C), and yield strength (70.52 MPa → 60.54 MPa/350 °C → 56.12 MPa/400 °C) compared to primary annealing.

(2) After primary annealing, the Cu-Cu joints exhibited an average grain size of 0.62 μm. With secondary annealing and an increase in annealing temperature (350 °C → 400 °C), the grain size of the Cu-Cu joints was further promoted to 0.67 μm and 0.71 μm, respectively. Concurrently, the kernel average misorientation (KAM) value decreased by up to 12%, indicating a significant reduction in dislocation density. These results reflect that the recrystallization–grain growth mechanism dominated the microstructural evolution during annealing. This process also enhanced the non-planarization of the Cu-Cu interface and optimized bonding quality.

(3) The secondary annealing treatment significantly enhances the mechanical properties of Cu-Cu joints by modulating its microstructure: increased grain size reduces hardness and yield strength in accordance with the Hall–Petch relationship, while residual stress release lowers the elastic modulus. Significantly, the yield strength of secondary annealed Cu-Cu joints reaches 56.12 MPa, demonstrating a 31.0% and 68.5% enhancement compared to TSV-Cu (42.83 MPa) and bulk Cu (33.3 MPa).

Hybrid bonding finds extensive application primarily in high-density interconnect electronic products (such as HBM), sharing similarities with TSV-Cu application scenarios. However, due to its process capability to achieve finer pitch (2–6 μm), it offers higher interconnect density. Following secondary annealing, the release of residual stress reduces the dislocation density within the Cu-Cu joints in hybrid bonding, leading to a decrease in yield stress. Nevertheless, its yield stress level remains substantially higher than that of TSV-Cu.

## Figures and Tables

**Table 1:** Power law constitutive parameters of Cu-Cu joints in different states.

State	Elastic Modulus, *E* (GPa)	$\mathrm{Yield}\;\mathrm{Strength},\;\sigma_{y}$ (MPa)	Hardening Exponent, *n*
300 °C	131.02 ± 3.41	70.52 ± 9.77	0.4975 ± 0.002
300 °C + 350 °C	119.13 ± 5.02	60.54 ± 2.66	0.5096 ± 0.001
300 °C + 400 °C	118.98 ± 4.75	56.12 ± 4.36	0.4961 ± 0.01

## Data Availability

The original contributions presented in this study are included in the article. Further inquiries can be directed to the corresponding author.
